# An Unusual Case of Mirizzi Syndrome With Double Spontaneous Gallbladder Fistulas With the Colon and the Duodenum Presenting As Acute Cholecystitis

**DOI:** 10.7759/cureus.15978

**Published:** 2021-06-28

**Authors:** Nikolaos Pararas, Abdulkarim M Alkadrou, Rahil L Sayed, Andreas Pikoulis, Emmanouil Pikoulis

**Affiliations:** 1 Surgery, Dr. Sulaiman Al-Habib Medical Group/Alfaisal University, Riyadh, SAU; 2 Plastic Surgery, AlMaarefa University, Riyadh, SAU; 3 Radiology, Dr. Sulaiman Al Habib Medical Group/Alfaisal University, Riyadh, SAU; 4 Third Department of Surgery, Attikon University Hospital, National & Kapodistrian University of Athens Medical School, Chaidari-Athens, GRC

**Keywords:** mirizzi syndrome, preoperative misdeception by radiology, surgical managment, double fistulas, synchronous cholecystoduodenal and cholecystocolonic fistulas

## Abstract

The Mirizzi syndrome is amongst the rarest complications of long-standing gallstone disease. It is an even rarer occurrence when concurrent with a cholecystoenteric or cholecystoduodenal fistula and might not include an accompanying gallstone ileus. Chronic cholecystitis is the primary etiology, but pre-operative diagnosis is challenging due to its non-specific symptoms compared with acute cholecystitis. In this unusual case, the patient has a Csendes type Va Mirizzi syndrome associated with a double cholecystoduodenal and cholecystocolonic fistula, a rare presentation.

## Introduction

The Mirizzi syndrome was first described and documented in 1948. Among surgeons is known to indicate the condition in which a large gallstone causes a narrowing of the common hepatic and common bile ducts in the gallbladder neck or the cystic duct [1]. Mirizzi syndrome is a rarer complication of chronic gallstone disease, along with gallstone ileus and cholecystocholedochal fistula [2]. When Mirizzi syndrome appears with a cholecystoenteric fistula, it is an even rarer occurrence and might or might not include an associated gallstone ileus [2,3]. A cholecystoenteric fistula is a spontaneous communication between the inflamed gallbladder and one or more sites of the adjacent gastrointestinal tract. Of these patients who have both Mirizzi syndrome and cholecystoenteric fistula, most of the studies reported cases with cholecystoduodenal fistula and cholecystogastric fistula.

On the other hand, there are limited reports for cases with cholecystocolonic fistula. Cholecystoduodenal fistulas represent approximately 75-80% of all cholecystoenteric fistulas with an overall incidence 0,5-0,9% [4-7]. The most common cause for as many as 75% of all cholecystoenteric fistula patients is chronic cholecystitis [7]. Pre-operative diagnosis is challenging due to cholecystoenteric fistula patients’ presentation with non-specific symptoms compared to cholecystitis [8]. 

In the following case, the patient had an advanced Csendes type Va Mirizzi with a double cholecystocolonic and cholecystoduodenal fistula, which were missed by the pre-operative investigations, and was found intraoperatively, representing a rare presentation of a rare case. Previously in the literature, only one case was reported and explained in detail, in 1951 by Lapeyere et al. [9].

## Case presentation

The patient is an otherwise healthy 51-year-old male patient with a known history of chronic biliary colic for two years that presented to the ER complaining of right upper quadrant abdominal pain for one week, which was worsened by fatty or spicy food intake, associated with two episodes of coffee ground appearance vomiting. He denied any fever, change in bowel motion, itchiness, discoloration of skin or sclera, no change in colour of urine or stool, and no history of generalized fatigue, weight loss, and night sweating. He was vitally stable, afebrile, with an optimistic Murphy’s sign and no rebound tenderness on physical examination.

**Figure 1:**
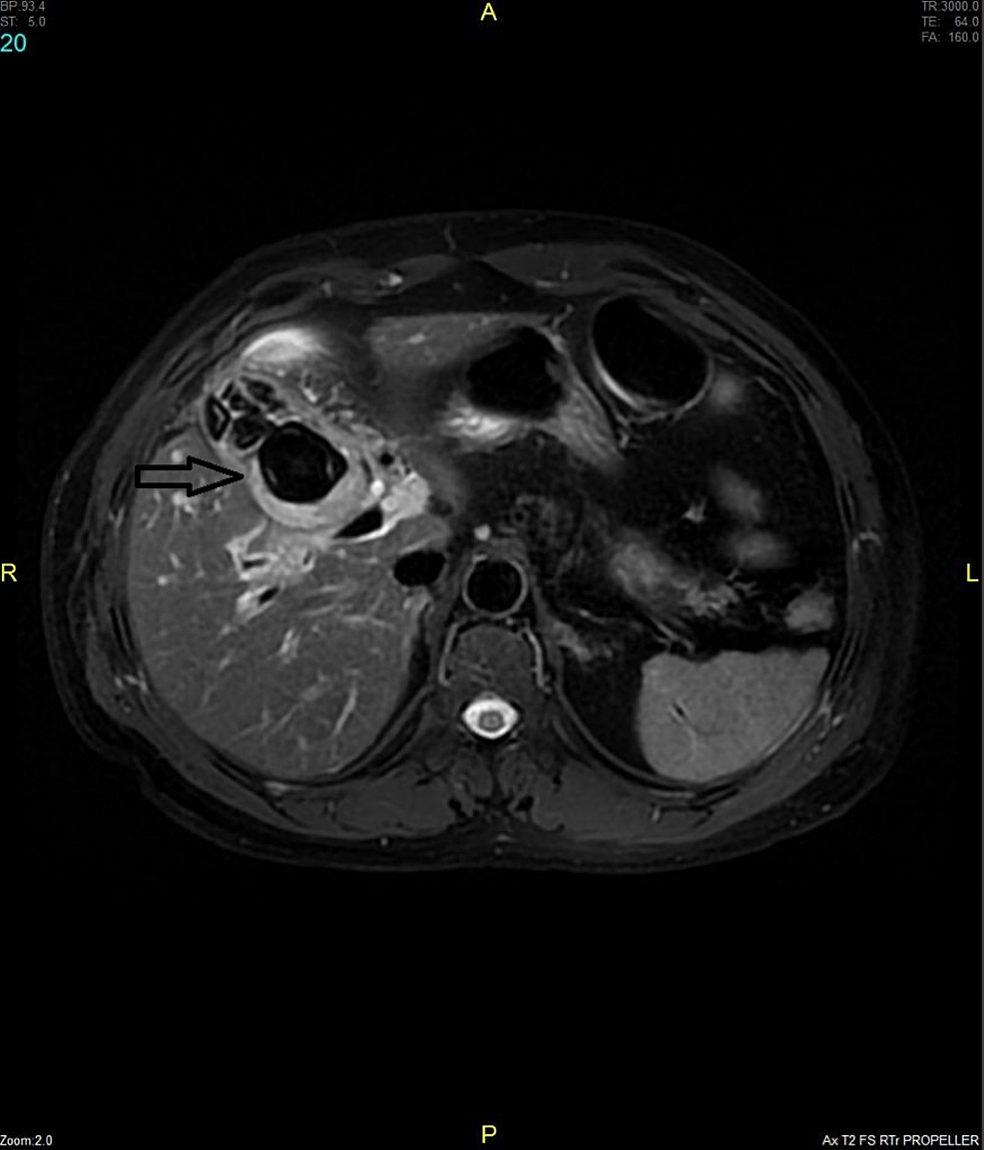
The figure shows a 2.9 x 2.7 cm impacted calculus at the neck of the gallbladder (arrow) with adjacent thickened gallbladder wall.

**Figure 2:**
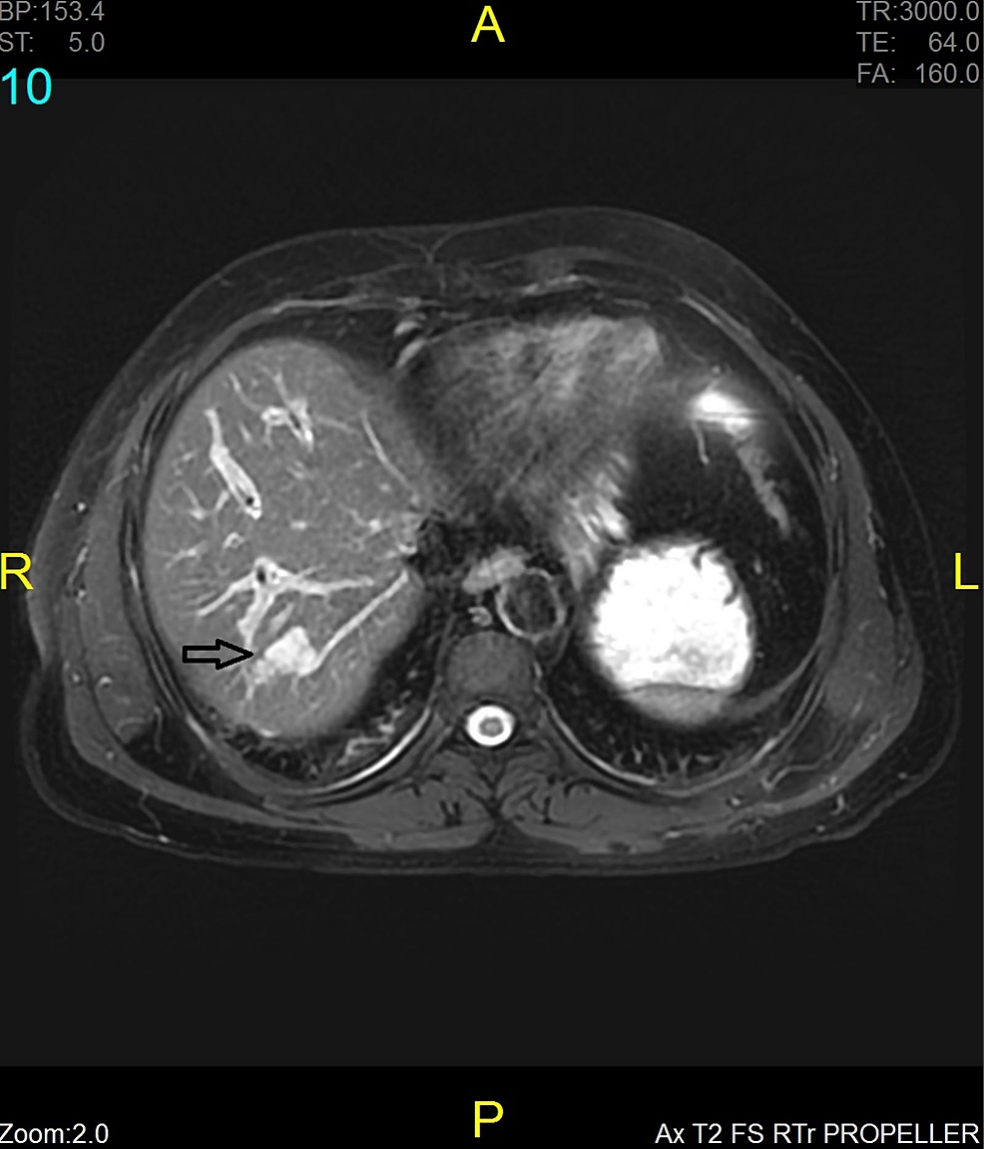
The figure shows a lobulated high signal intensity 2.4x 1.4cm hemangioma (arrow) in segment 7 of the liver.

Upper abdominal Ultrasound showed that the liver was normal in size and echotexture, with no focal lesion and the intrahepatic ducts not dilated, and no evidence of ascites. The gall bladder (GB) was not delineated, and dense posterior acoustic shadowing was noted in the GB fossa. The Common bile duct and portal vein appeared normal. Laboratory tests revealed slight leucocytosis with white blood cells (WBC)- 10,150/μl, haemoglobin (Hgb) 2.4 gr/dl, and elevated liver function tests as follows: alkaline phosphatase (ALP)- 283 IU/lt (normal value 40-150 IU/lt), aspartate aminotransferase (AST)- 366 IU/lt ( normal values 5-32 IU/lt), alanine aminotransferase (ALT) 427 IU/lt ( normal values 5-33 IU/lt), total bilirubin 32 μmol/lt ( normal values 3-21 μmol/lt), direct bilirubin 23.3 μmol/lt ( normal values 0.8-6 μmol/lt), and lipase and amylase within normal range. The patient was admitted and started resuscitation with intravenous fluids, he was kept nil per mouth, and a broad-spectrum antibiotic was started. A magnetic resonance cholangiopancreatography (MRCP) was done and reported as a distended gallbladder with multiple signal void areas representing cholelithiasis. A large, rounded signal void area near the neck of the gallbladder measuring 2.9 x 2.7cm with adjacent thickened gallbladder wall with edematous changes most likely represents impacted calculus at the neck of the gallbladder (Figure 1). It compressed the adjacent common hepatic duct with mild prominence of right and left hepatic ducts near the porta hepatis. Common bile duct measured 0.5 cm with no filling defect or stricture seen and no evidence of choledocholithiasis. The pancreatic duct also showed no definite stenosis or calculus. The liver appeared normal in size with smooth margins. A lobulated high signal intensity 2.4x 1.4cm lesion in segment 7 of the liver was a hemangioma (Figure 2).

As the MRCP was negative for any CBD stones or obstruction and the dilatation of the hepatic ducts was attributed to the pressure of the gallbladder to the common hepatic duct, we did not seek ERCP and proceeded for laparoscopic cholecystectomy.

**Figure 3:**
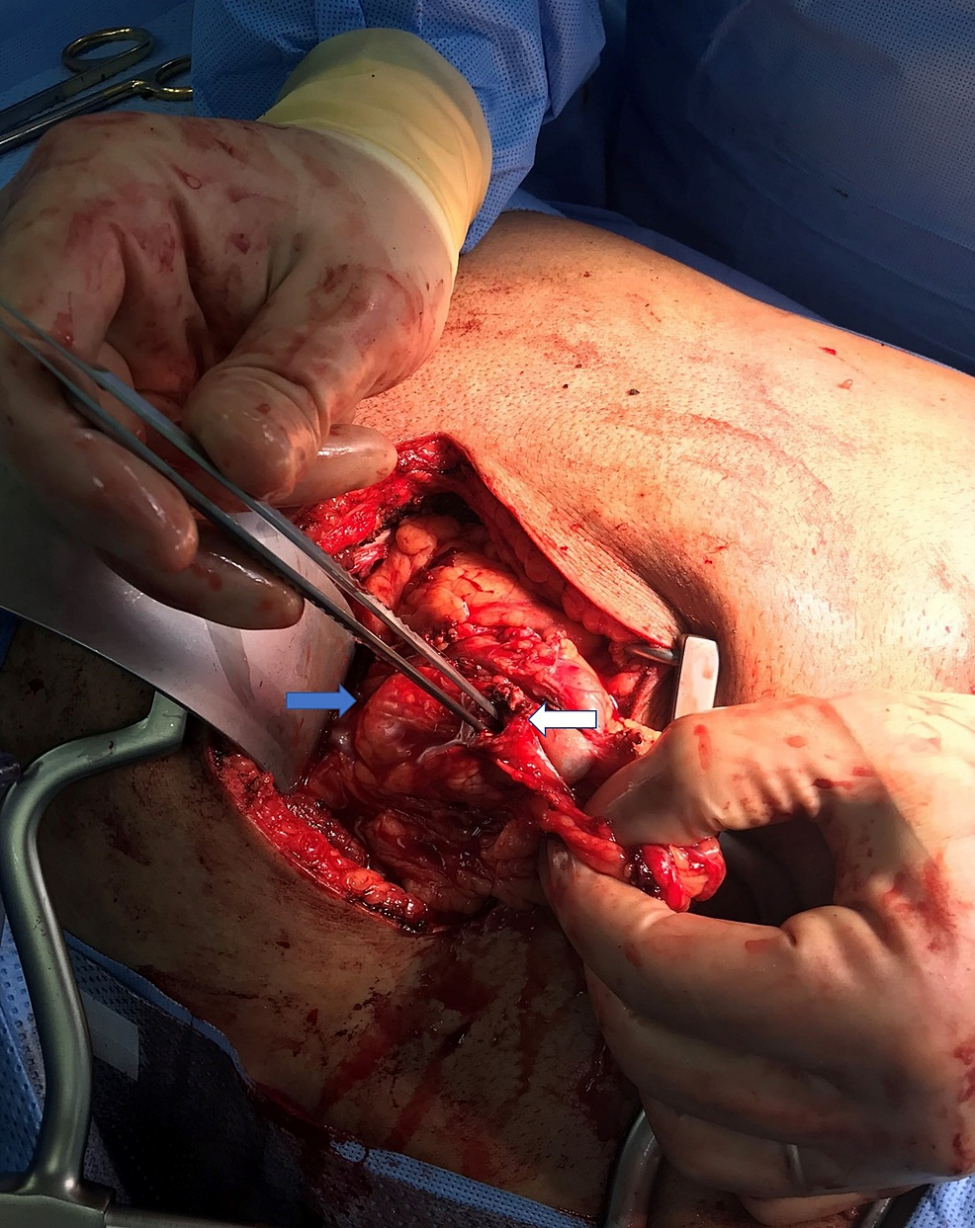
Cholecystocolonic fistula with the hepatic flexure. Blue arrow shows the fundus of the gallbladder and white arrow the fistula of the hepatic flexure.

**Figure 4:**
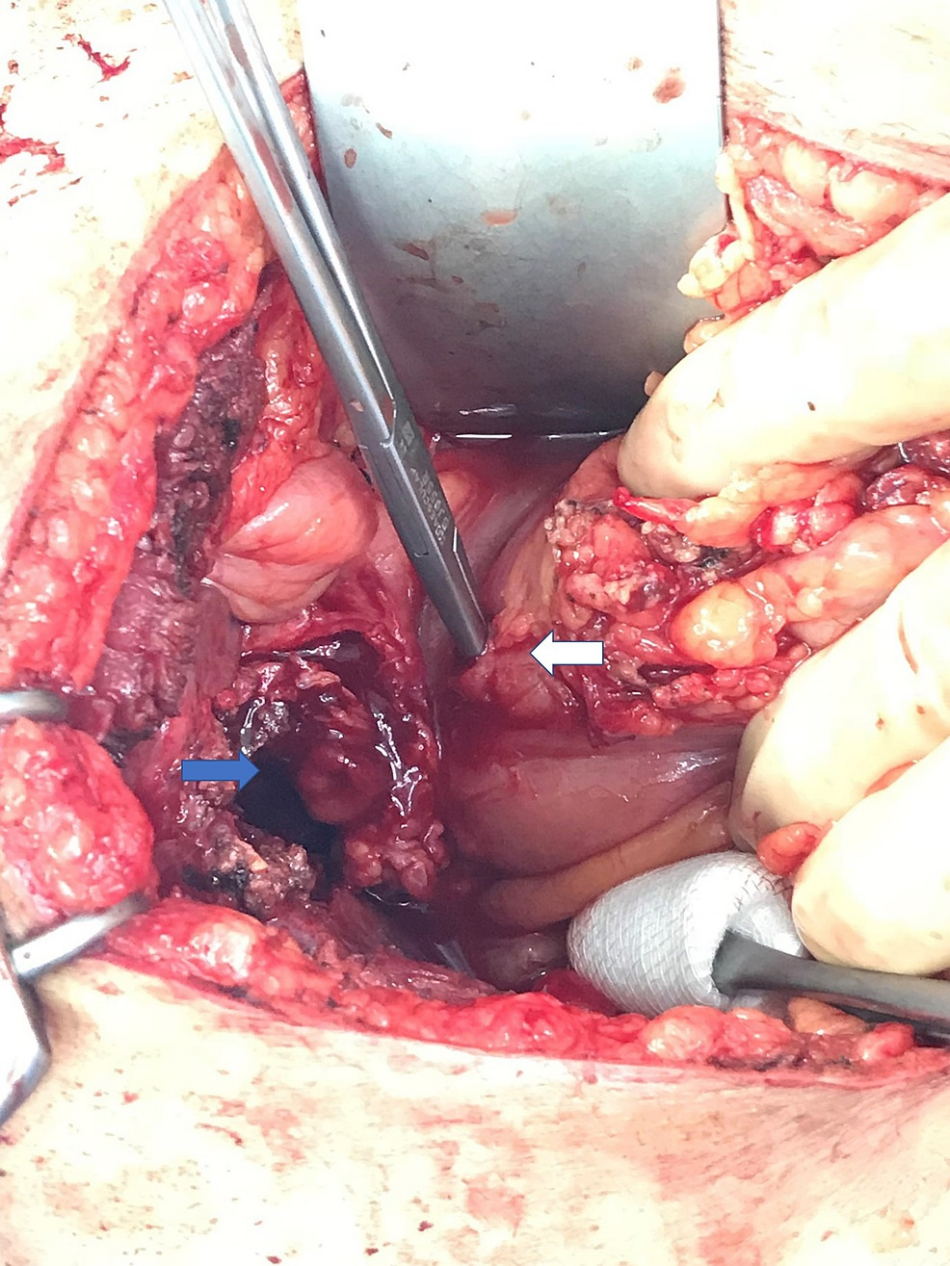
Cholecystoduodenal fistula. Blue arrow shows the partially excised gallbladder and the cystic duct and white arrow shows the fistula with the 1st part of the duodenum

The following day, the patient was booked for laparoscopic cholecystectomy. However, intraoperatively extensive dense adhesions were found in the right upper quadrant between the gallbladder, the colon, and the omentum, which was entirely adherent to the gall bladder. The anatomy was distorted was decided to convert to open given the patient’s safety. After a Kocher right subcostal incision was performed, and upon entering the peritoneum, the gall bladder fundus was found to have a fistulous communication with the hepatic flexure (Figure 3). The colon was divided from the gallbladder, and the fistula tract was en-block excised, and the colon defect was repaired in two layers. After that, the mobilization of the gallbladder continued, and upon reaching the Hartmann’s pouch, we found a 2nd fistula of the gallbladder with the 1st part of the duodenum in the anterior wall (Figure 4). The fistulous tract was again excised. The duodenum defect was sutured in the first intention as it was occupying almost 10% of the duodenal wall and an omental Graham’s patch sutured over the repair. Consecutively, a partial cholecystectomy was performed, as the gallbladder was very adherent to the common bile duct (CBD) but without invading it and no anatomical defects. A negative pressure drain was positioned in the gallbladder fossa, and the abdominal incision closed in the usual way. 

The postoperative period was uneventful, and after a methylene blue leak test was performed on a postoperative day four and oral initiated, which was negative, the patient was discharged on postoperative day six. On the 1st postoperative follow-up after seven days after discharge, he was stable and doing well and had returned to his everyday life. On follow-up one month postoperatively, all his laboratory investigations were back to normal, and the histopathology confirmed the presence of both fistulas without evidence of malignancy.

## Discussion

Mirzzi syndrome with cholecystoenteric fistula(s) is of the rare complications of chronic cholelithiasis [2]; the cholecystoenteric fistula occurs from chronic inflammation in the gallbladder wall, leading to necrosis of the gallbladder wall and erosion of adjacent tissues [10,11], as Courvoisier said in the early 1890s. The occurrence of a fistula with Mirzzi syndrome has demonstrated to relate to the severity of the syndrome [3]. In the Mirzzi syndrome, we usually use both Csendes and McSherry classification systems [1]; McSherry classified Mirzzi syndrome into two types: As type I is described an external compression to the bile duct by a large stone and as type II a cholecystobiliary fistula caused by a stone or stones [12]. Csendes offers further information to the surgeon with his classification related to the severity of Mirrzzi syndrome, making it more useful during the management of such patients. It classifies the Mirizzi syndrome into five Types: First, in type I, extrinsic compression to the common hepatic duct (CHD) is present. Second, it subdivides it to type Ia in case of an impacted gallstone in the cystic duct or gallbladder neck and type Ib if the cystic duct is absent. In Type II, he describes an erosion of the common hepatic duct wall and the formation of a cholecystocholedochal fistula (when up to one-third of the common hepatic duct wall circumference is involved). Type III involves up to two-thirds of common hepatic duct wall circumference in a cholecystocholedochal fistula. Type IV is when the entire common hepatic duct wall is involved in a cholecystocholedochal fistula. Csendes type V is suggestive of a cholecystoenteric ﬁstula in addition to any of the other types. Type V is then divided into type Va which includes patients without a gallstone ileus, and type Vb, including those with associated gallstone ileus.

As the diagnosis may be missed pre-operatively and the surgical treatment of this uncommon condition is related to an increased risk of a biliary injury, this classification is essential to the surgeon. Additionally, intraoperative recognition of such conditions can be complex, especially if a fistula is present or extensive adhesions complicate the dissection. Hence, a precise definition of the biliary anatomy pre-operatively is paramount for ideal surgical planning.

**Figure 5:**
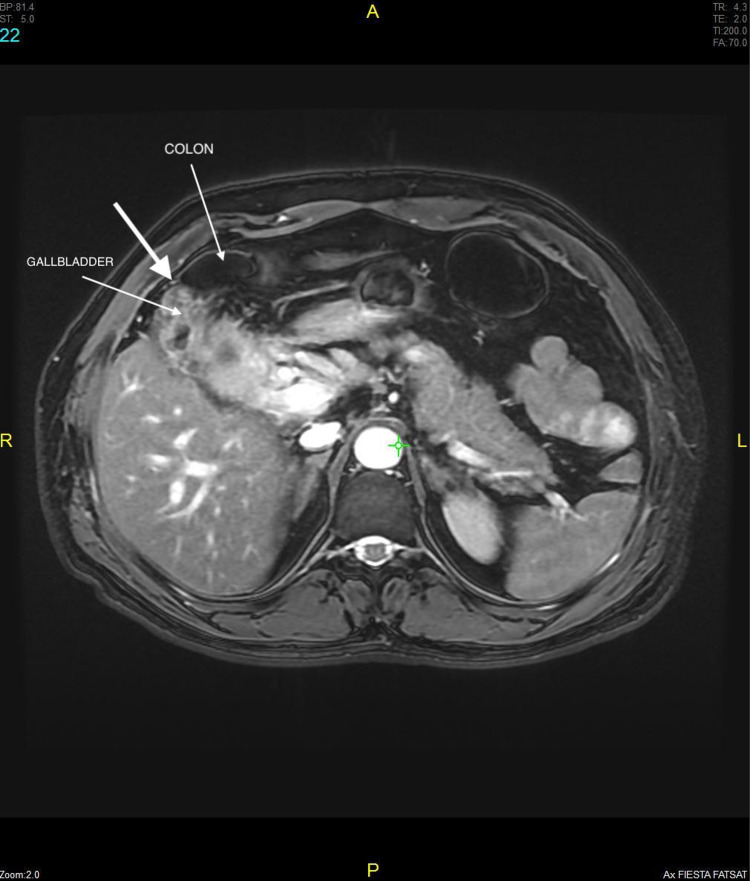
Axial image shows inflamed GB walls with intraluminal gallstones and fistulous communication with hepatic flexure of colon.

**Figure 6:**
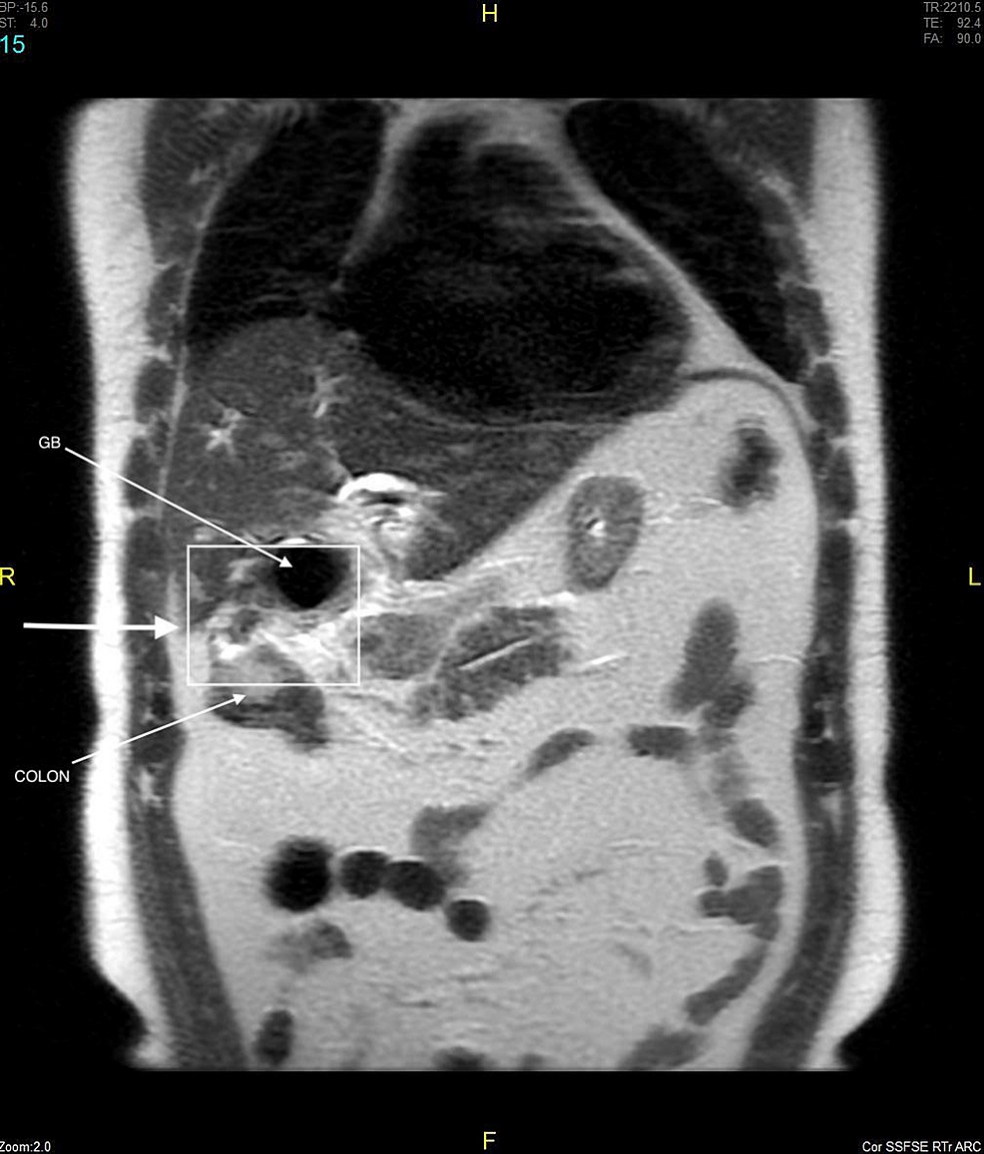
Coronal sequence image shows fistulous tract communicating inflammed GB with hepatic flexure of colon.

**Figure 7:**
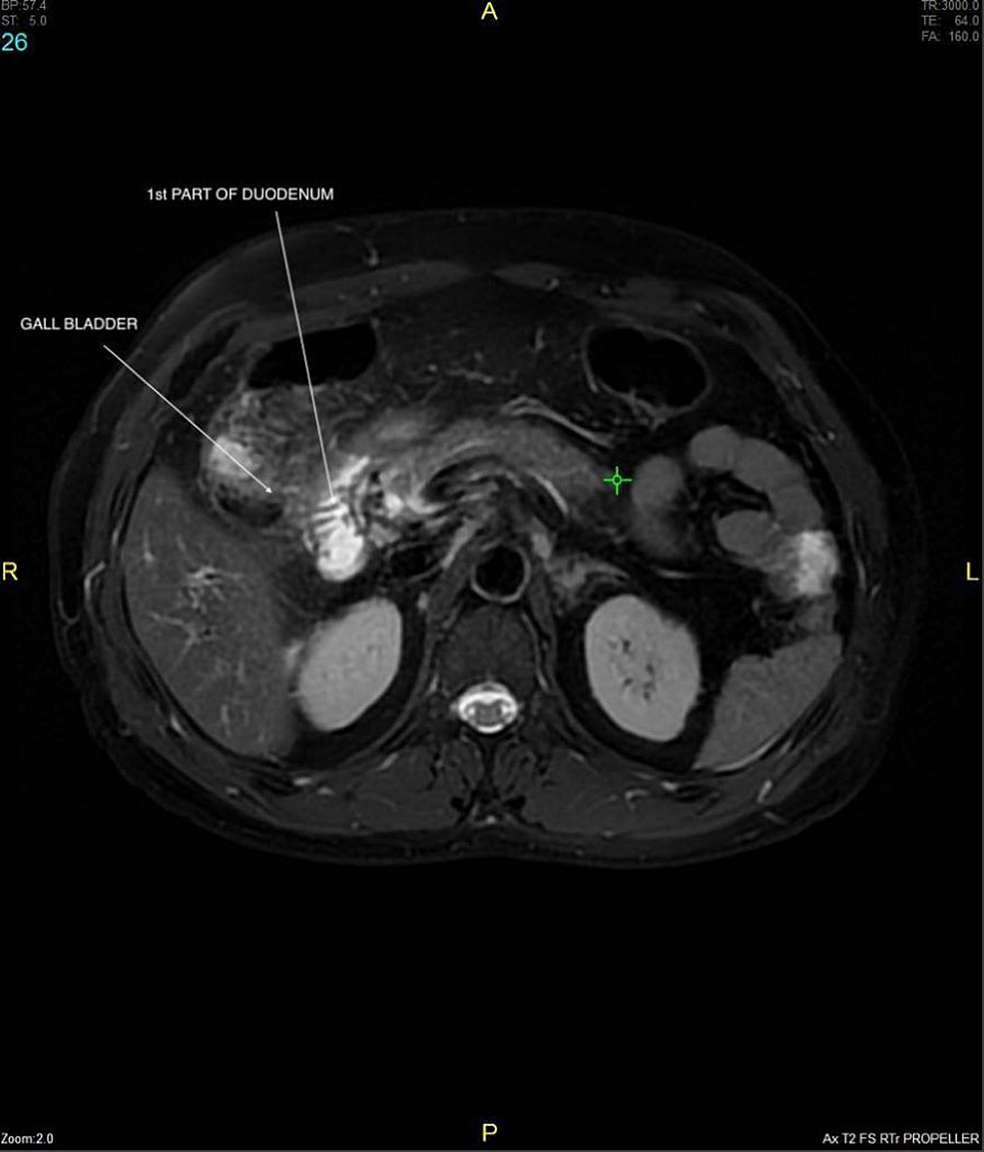
Axial sequence image shows inflamed GB walls with intraluminal gallstones and fistulous communication with 1st part of duodenum with adjacent duodenal wall thickening.

**Figure 8:**
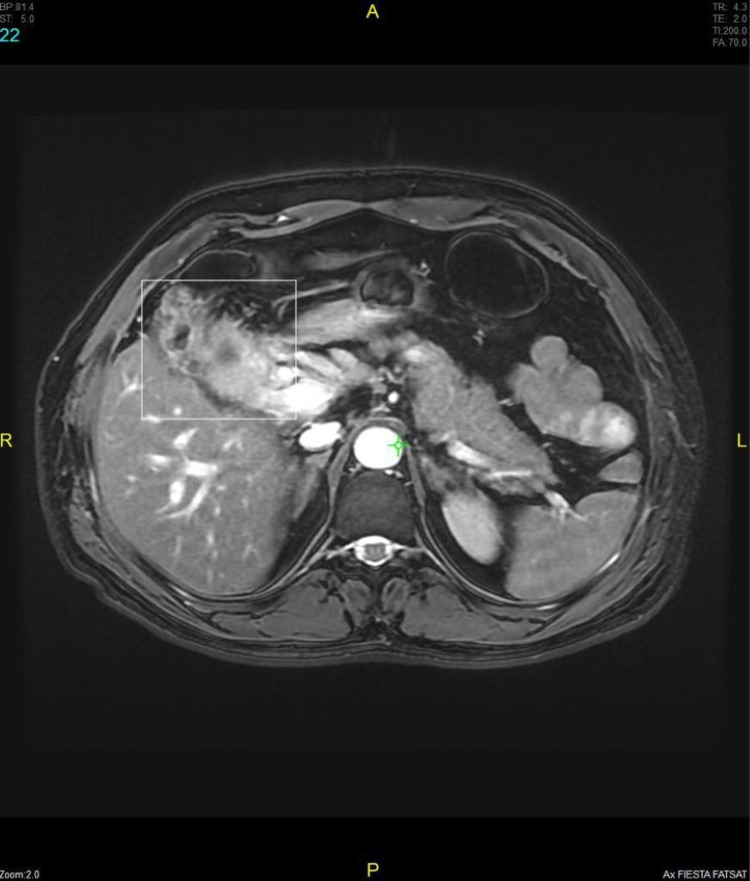
Axial sequence image shows inflamed GB walls with intraluminal gallstones and fistulous communication with 1st part of duodenum (quadrangle) with adjacent duodenal wall thickening.

**Figure 9:**
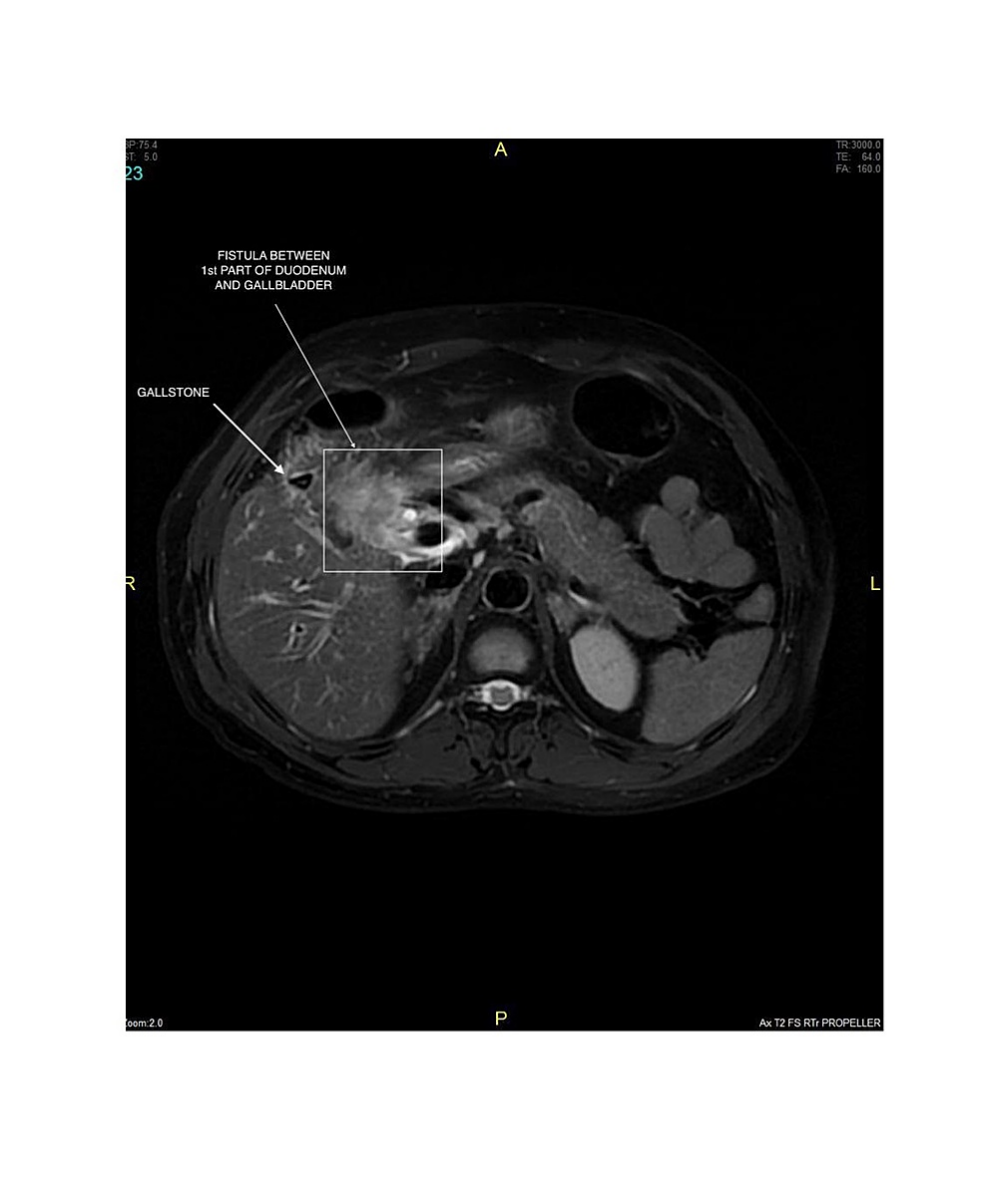
Axial sequence image shows inflamed GB walls with intraluminal gallstones and fistulous communication with 1st part of duodenum with adjacent duodenal wall thickening.

Our patient was type Va, which was discovered intraoperatively. Despite having two fistulas in two different areas (colon and duodenum), he did not change bowel habits or obstruction. About 3-5% of patients develop single fistulas between the extrahepatic biliary and gastrointestinal systems [13]. However, it is a rare and unusual complication to find two spontaneous fistulas with two different sites as in our patient. The most common site of a fistula formation is the gallbladder and one of the adjacent viscera due to chronic inflammation and impaction of stone at the ampulla or in the cystic duct. The patient with a fistula usually presents with a clinical picture of chronic cholecystitis and sometimes with peptic ulcer symptoms or symptoms of malignancy if the fistula is found to be with the duodenum. Our patient, who presented with coffee ground vomiting, was diagnosed intraoperatively with cholcystoduodenal fistula, which explained his symptoms and bile irritation of the duodenum [14,15]. The cholecystoenteric fistula is easy to miss or misdiagnose because the patient will present with a clinical picture of chronic cholecystitis. The imaging can underestimate the extent of the disease, as happened in our patient. The pre-operative MRCP did not show any of the fistulas and deceived us as per the diagnosis. A postoperative review of the MRCP with the radiologists, after we knew the correct diagnosis of the double fistulas, made it clear that the fistula tracts were visible in the MRCP but misinterpreted as changes of chronic cholecystitis (Figures 5-9).

It is difficult to find a Mirizzi’s syndrome in a patient, and it is difficult and not so common to diagnose pre-operatively. This indicates that this syndrome is not associated with unique clinical features or a precise set of demographics. For example, the patient in our case was 51 years old. Additionally, he presented with laboratory and clinical findings generally consistent with acute cholecystitis with possible obstruction of the common bile duct.

The absence of a consistent clinical presentation poses a significant challenge for surgeons and primary care physicians. Due to the limitations above, a pre-operative diagnosis of Mirizzi syndrome depends upon the help of imaging investigations. Plain abdominal X-Rays alone are not effective. Likewise, Ultrasound (US) or CT scans are not often diagnostic. However, both US and CT scan studies may reveal findings that strongly suggest the diagnosis, such as (1) dilatation of the biliary system above the level of the gallbladder neck, (2) an impaction of a stone or stones in the gallbladder neck, and (3) a common bile duct (CBD) of normal calibre below the impaction level. In addition, recent studies suggest that Magnetic Resonance Cholangiography (MRCP) can be valuable in diagnosing Mirizzi syndrome [16].

Additionally, Wehrmann reported the use of intraductal ultrasonography to diagnose Mirizzi syndrome in 30 patients with a specificity of 100% and a sensitivity of 97% [15]. First, we should consider that the findings observed in the imaging studies associated with Mirizzi syndrome may not be distinguishable from those of ductal and periampullary malignancy. A good clinical history and examination and the presence of adenopathy or portal or hepatic masses may help to differentiate these two different entities.

Mirizzi’s syndrome treatment continues to be challenging and must be tailored to the stage of the disease and depends on the expertise and experience of the surgeon. The stone, which is obstructing, is commonly not retractable by endoscopy, and for this reason, the definitive treatment of Mirizzi Syndrome remains surgical [17]. Surgical intervention should meet three objectives: Extract the obstructing stone, remove the gallbladder, and restore normal biliary flow and drainage. In general, the choice of the specific operative technique depends on a precise definition of the biliary anatomy. Csendes et al. classified the Mirrizzi syndrome into five types to better guide this surgical management and showed in their study of 219 patients that significantly higher rates of postoperative morbidity and mortality were associated with the more severe grades of the disease [8]. Our patient was a type Va Csendes Mirrizzi syndrome with two fistulas. However, the patient was successfully managed with minimal damage to the bowel with only fistula tract excision and bowel repair with primary closure without any bowel resection.

## Conclusions

The Mirizzi syndrome remains a rare but significant cause for obstructive jaundice and poses significant challenges to the surgeon. Non-invasive laboratory investigations and imaging studies provide supportive diagnostic information and sometimes are not enough for a definite diagnosis. Therefore, a pre-operative Endoscopic retrograde Cholangiopancreatography (ERCP) or Percutaneous Transhepatic Cholangiography (PTC) may be necessary to determine the diagnosis delineate the anatomy and achieve decompression of the obstructed ductal system pre-operatively. Even So, even ERCP is not always effective and successful. Therefore, an operation tailored to the specific clinical and anatomical findings is required as definitive treatment. Available surgical options range between partial or total cholecystectomy and more complex biliary-enteric anastomoses and choledochoplasties. Attaining the best results depends on a specialized, multidisciplinary team experienced in managing advanced biliary diseases.
